# Evaluation of the Bioaccessible Fraction of T-2 Toxin from Cereals and Its Effect on the Viability of Caco-2 Cells Exposed to Tyrosol

**DOI:** 10.3390/toxins15080493

**Published:** 2023-08-04

**Authors:** Carmen Martínez-Alonso, Mercedes Taroncher, Yelko Rodríguez-Carrasco, María-José Ruiz

**Affiliations:** Department of Preventive Medicine and Public Health, Food Science, Toxicology and Forensic Medicine, Faculty of Pharmacy, University of Valencia, Burjassot, 46100 Valencia, Spain; carmen.martinez-alonso@uv.es (C.M.-A.); mercedes.taroncher@uv.es (M.T.); m.jose.ruiz@uv.es (M.-J.R.)

**Keywords:** bioaccessibility, in vitro digestion, T-2 toxin, UHPLC-MS/MS, viability

## Abstract

The bioaccessibility of mycotoxins is an important factor that has to be considered when assessing the risk they pose to human health. Bioactive compounds like phenolics could play a protective role against the toxic effects of contaminants. In this work, the bioaccessible fraction of the T-2 toxin (T-2) contained in breakfast cereals and its effect on the viability of Caco-2 cells were investigated. Furthermore, the effect of tyrosol (a polyphenol abundant in EVOO) on T-2-induced cytotoxicity was evaluated in the same cell line. After standardized in vitro gastrointestinal digestion, the T-2 toxin was released from T-2-spiked breakfast cereals and further quantified by UHPLC-MS/MS. The bioaccessible fraction of T-2 was 51 ± 4%. The cell viability study was performed by pre-treating the cells for 24 h with tyrosol (25, 50 and 100 µM) and subsequently adding T-2 at 15 nM or by treating the cells with a combination of tyrosol and T-2. In the simultaneous treatment, 25 µM tyrosol prevented the toxic effects produced by the exposure to T-2 at 15 nM; however, cytotoxic effects were observed for the other combinations tested. The pre-treatment of Caco-2 cells with tyrosol did not attenuate the cytotoxic effects caused by exposure to T-2. These results suggest that tyrosol at low concentrations (25 µM) could exert a cytoprotective effect on Caco-2 cells against 15 nM T-2 when administered simultaneously with T-2. However, more studies are required to corroborate this hypothesis.

## 1. Introduction

Cereals and cereal-based products stand out as a source of energy and fiber whose intake has been related to multiple benefits at the intestinal, metabolic and disease prevention level [1]. Despite the relevant role of this group of foods in human nutrition, cereals are susceptible to natural contamination by fungi that can produce toxic substances under certain environmental conditions, known as mycotoxins [2].

Mycotoxins are a wide and heterogeneous group of toxic secondary metabolites produced by different species of filamentous fungi that can contaminate food and feed and result in a significant threat to human and animal health. The main mycotoxin-producing fungi belong to the genera *Aspergillus*, *Penicillium*, *Fusarium*, *Claviceps* and *Alternaria* [3]. These toxins are among the natural contaminants in food of greatest concern from a public health point of view. In fact, mycotoxins represent the third most notified hazard category, with a 6% increase compared to 2020, as evidenced in the latest Rapid Alert System Feed and Food (RASFF) publications [4]. Trichothecenes are a complex group of tetracyclic sesquiterpenoids produced by several *Fusarium* spp. The T-2 toxin (T-2) is the compound with the highest toxicity within the group of trichothecenes [5]. Some studies reported that T-2 can induce reactive oxygen species (ROS) accumulation, an increase in malondialdehyde (MDA) level and a reduction in the activities of superoxide dismutase (SOD), catalase (CAT) and glutathione peroxidase (GSH-Px), resulting in oxidative stress [6]. The toxic effects derived from repeated exposure to T-2 include genotoxicity, immunotoxicity, neurotoxicity and reproductive toxicity [7]. Based on the toxic effects produced by T-2 and its frequent occurrence in food, indicative levels were set for T-2 and the HT-2 toxin, its main metabolite, from 75 to 1000 μg/kg in cereals and cereal-based products [8]. In addition, the European Food Safety Authority (EFSA) established a tolerable daily intake (TDI) for the sum of T-2 and HT-2 of 0.02 µg/kg bw to limit their exposure [5].

On the other hand, Extra Virgin Olive Oil (EVOO), the main fatty constituent of the Mediterranean diet, has been associated with a general health benefit and a minor incidence of risk factors for coronary heart disease. This is probably owing to its high amount of monounsaturated fatty acids and to the presence of phenolic compounds, which have a strong antioxidant activity [9,10]. The long-term intake of phenolic compounds could play a bioactive role, promoting an antioxidant effect associated with the prevention of cardiovascular and neurodegenerative diseases, diabetes and cancer [11,12]. The antioxidants present in EVOO are capable to scavenge free radicals and provide an appropriate protection against peroxidation [13]. On the other hand, the consumption of EVOO has shown positive effects on the gut microbiota and intestinal health, promoting the growth of beneficial bacteria. The presence of phenolic compounds in EVOO is probably responsible for the capability of EVOO to act like an antibacterial (suppressing the growth of pathogenic bacteria) and a prebiotic (incentivizing the growth of beneficial bacteria) [14]. The main antioxidant group in EVOO consists of polyphenols, being tyrosol one of the most representative compounds. Recently, the bioaccessibility and stability of phenolic compounds from EVOO using the INFOGEST standardized in vitro gastrointestinal method has been proven [15]. The toxic effects of T-2 are conditioned by its bioaccessibility, bioavailability and metabolic fate. In vitro static methods simulating gastrointestinal digestion have been widely applied to predict the bioaccessibility of several mycotoxins [16]. Bioaccessibility means the capability of toxic compound released from a food matrix to pass across the intestinal barrier. Since the bioavailability of T-2 depends on its digestive stability and release from food matrixes, the bioaccessibility of this mycotoxin should be investigated. To evaluate the cytotoxic effect produced by mycotoxins, in vitro techniques are extensively used [17]. Similarly, cell culture techniques are also employed to study the cytoprotection effect of polyphenols in cells exposed to mycotoxins [18]. Regulatory agencies recognized monolayers of epithelial cells from the human colon (Caco-2) as a standard model to investigate the effects of drugs or other toxic substances because of the good correlation observed between data on oral absorption in humans and results in Caco-2 cells [19]. In vitro models using Caco-2 cells have been employed for evaluating the bioaccessibility, permeability, bioavailability and intestinal transport of toxic compounds, including mycotoxins [20].

Based on the above-mentioned information, the purpose of this investigation were (i) to determine the bioaccessibility of T-2 contained in breakfast cereals through the INFOGEST standardized in vitro gastrointestinal digestion and to further quantify it with ultra-high-performance liquid chromatography coupled to tandem mass spectrometry (UHPLC-MS/MS), (ii) to assess the exposure effect of the bioaccessible fraction of T-2 in Caco-2 cell viability and (iii) to evaluate the effect of tyrosol on T-2-induced cytotoxicity in Caco-2 cells.

## 2. Results and Discussion

### 2.1. Bioaccesibility Fraction of T-2

Firstly, the assessment of the bioaccessible fraction of the T-2 toxin from breakfast cereals was carried out by the INFOGEST standardized in vitro gastrointestinal digestion, followed by the toxin quantification with UHPLC-MS/MS. T-2 released from breakfast cereals during the simulated gastrointestinal digestion was evaluated in the control (blank samples) and spiked breakfast cereals by the analysis of oral, gastric and intestinal extracts. The digestion of the spiked samples was performed in triplicate. Table 1 presents the results.

During gastrointestinal digestion, the amount of T-2 released from the food matrix was in the range from 25% to 58%. These values may be due to its binding to long-chain carbohydrates, abundant in cereals, which are hydrolyzed in duodenal digestion. A similar trend was recently reported in the literature, attributing the fluctuation of the bioaccessible fraction of mycotoxins (aflatoxin B1 and ochratoxin A) in bread to possible interactions between the food matrix, the mycotoxin and the digestive fluids in each phase [21].

**Figure 1:**
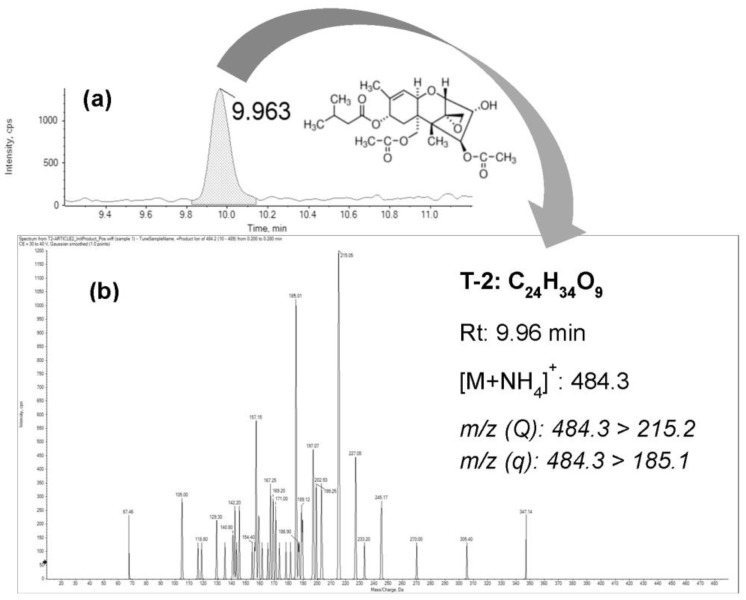
(**a**) UHPLC-MS/MS chromatogram of a digested (intestinal phase) breakfast cereal sample spiked with T-2 at 75 µg/kg; (**b**) mass spectrum of T-2 after fragmentation of the precursor ion [M + NH_4_]^+^: 484.3.

Based on the here-reported data, a bioaccessible fraction of T-2 of 51 ± 4% was obtained in the last step of the in vitro digestion (Table 1). The obtained values are in accordance with De Angelis et al. [22], who showed a bioaccessible fraction for T-2 of 52 ± 9% after the in vitro digestion of bread spiked with the toxin and evaluated by UHPLC-MS/MS. Figure 1 shows the chromatogram of the bioaccessible fraction of T-2 from breakfast cereals spiked with this toxin at 75 µg/kg.

Regarding the bioaccessible fractions of mycotoxins other than T-2, Raiola et al. [23] determined the bioaccessibility of deoxynivalenol in pasta samples by an in vitro digestion model and further quantified it by liquid chromatography tandem mass spectrometry (LC-MS/MS), obtaining values between 1.1 and 24.1%. Kabak et al. [24]. reported bioaccessibility values of aflatoxin B1 and ochratoxin A in artificially contaminated baby foods between 88 and 94% and between 29 and 32%, respectively. However, in this work a significant reduction in the bioaccessibility of aflatoxin B1 and ochratoxin A was observed in the presence of probiotic bacteria. The differences observed between the bioaccessibility values may be due to several factors such as the food matrix, the mycotoxin, the concentration of the toxin and the type of contamination (natural or artificial). Hence, bioaccessibility depends on the mycotoxin and the food matrix considered. The bioaccessible values of mycotoxins reported in the literature and also those found in this study are of high interest from a toxicological perspective, because a significant percentage of mycotoxin can be able to exert toxic effects after its absorption through the intestinal epithelium. This implies that for risk assessment studies, it would be convenient to obtain specific results on the bioaccessibility of mycotoxins to adjust the maximum limits of mycotoxins in the different food groups, as argued in the review by González-Arias et al. [17].

### 2.2. Effect of the Bioaccessible Fraction on Cell Viability

**Figure 2:**
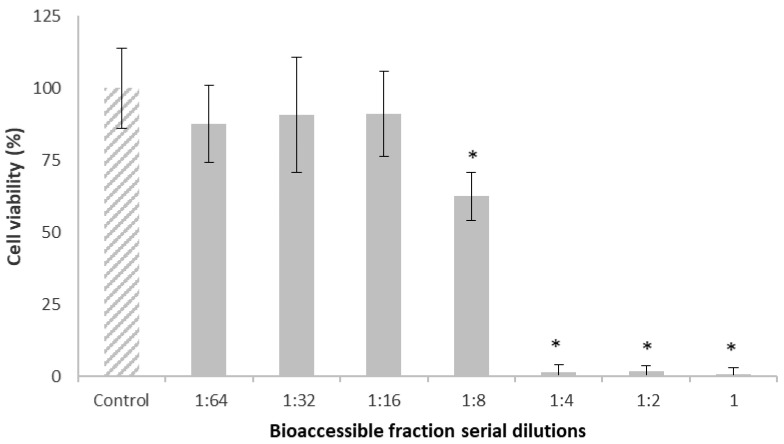
Caco-2 cells viability (%) after 24 h exposure to the bioaccessible fraction of T-2 from breakfast cereals, evaluated by the MTT assay. Serial dilutions of the bioaccessible fraction (from 1:0 to 1:64) were obtained with distilled water. All values are expressed as mean ± SEM of 3 replicates. (*) *p* ≤ 0.05 indicates significant differences compared to control cells (medium with a final DMSO concentration ≤ 1%).

Figure 2 shows the results of Caco-2 cell viability evaluated by the methylthiazoltetrazolium salt (MTT) assay after 24 h of exposure to the bioaccessible fraction of T-2 from breakfast cereals. As digestion enzymes are themselves toxic to cells, several dilutions were performed (from 1:0 to 1:64). The results plainly showed that the more concentrated dilutions affected Caco-2 cell viability. Particularly, the undiluted (1) and diluted (1:2, 1:4 and 1:8) bioaccessible fraction significantly decreased (*p* ≤ 0.05) Caco-2 cell viability.

At the dilution of 1:16, cell viability was not affected; it was similar to that of the control cells (Figure 2). So, this dilution was chosen for evaluating Caco-2 cell viability after exposure to the bioaccessible fraction spiked with T-2 at 75 µg/kg, because it was the most concentrated dilution that by itself did not significantly decrease cell viability with respect to the control.

**Figure 3:**
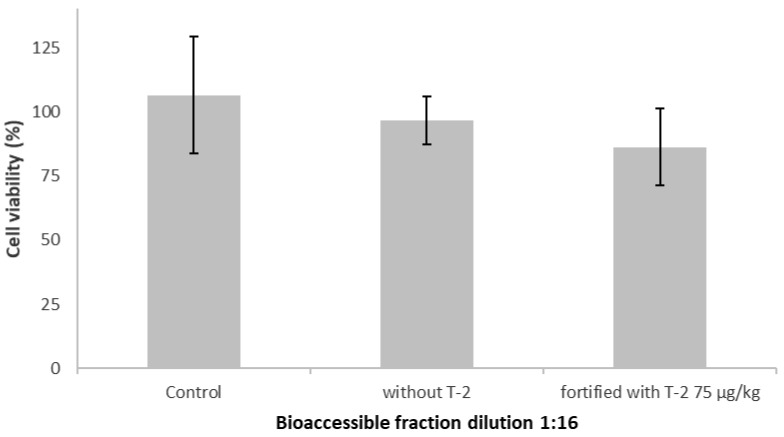
Caco-2 cells viability (%) after 24 h of exposure to the bioaccessible fraction (dilution 1:16) spiked with/without T-2 toxin in comparison with that of control cells (reagent blank including enzymes) evaluated by the MTT assay. All values are expressed as mean ± SEM of 3 replicates.

The results showed that the bioaccessible fraction of T-2 contained in the 1:16 dilution obtained from spiked samples did not affect cell viability (Figure 3). This could be justified based on the concentration of T-2 present in the wells. Taking into account the initial spiking level (75 µg/kg), the amount of initial sample taken for the in vitro digestion process (2.5 g), the concentration factor, linked to the complete digestion and subsequent extraction of the analyte (CF = 0.48), the bioaccessible fraction dilution (1:16) and the final volume of the well (200 µL), the final concentration of T-2 in the well was 4.82 nM. Thus, these results indicated that the T-2 concentration in the wells would not cause changes in Caco-2 cells viability.

However, individual exposure to T-2 toxin could be much higher considering other important food matrices contaminated by T-2, and therefore the total diet should be taken into consideration, as recommended when performing risk assessment studies. In fact, contamination by T-2 of other widely consumed cereals (such as oat, corn, barley, rice and beans) and its derived products has been reported in the literature. [5]. Based on that, to complete this investigation and move experiments closer to reality, Caco-2 cells were exposed to higher concentrations of T-2 by using an analytical standard.

### 2.3. Effect of T-2 on Caco-2 Cells’ Viability

**Figure 4:**
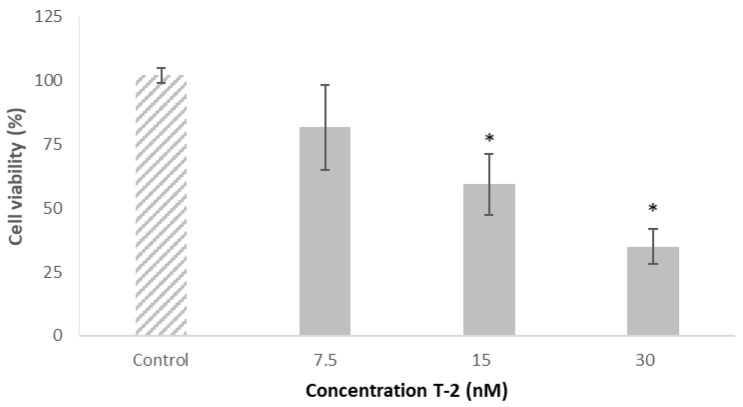
Concentration–cell viability (%) relationship in response to T-2 (7.5, 15 and 30 nM) in Caco-2 cells after 24 h of exposure, evaluated by the MTT assay. All values are expressed as mean ± SEM of 3 replicates. (*) *p* ≤ 0.05 indicates significant differences compared to control cells (medium with a final DMSO concentration ≤ 1%).

The assayed T-2 concentrations were based on previous studies carried out in the same cell line in which the half-maximal inhibitory concentration (IC_50_) value was reported [25]. Based on that, the T-2 concentrations of 7.5, 15 and 30 nM were used. The effect on the viability of Caco-2 cells exposed to T-2 at various concentrations (7.5, 15 and 30 nM) is shown in Figure 4. Caco-2 cells’ viability was decreased in a concentration-dependent manner. T-2 at 15 and 30 nM significantly decreased cell viability with respect to that of control cells, by 43% and 67%, respectively, thus showing a cytotoxic effect in this cell line. Otherwise, T-2 at the lowest concentration tested (7.5 nM) did not significantly affect cell viability with respect to the control. These results support the findings reported in Section 2.2. The T-2 concentration of 15 nM was chosen for further studies, due to the fact that it significantly affected Caco-2 cell viability, but this concentration was above the IC_50_ value.

Bouaziz et al. [26] evaluated the cell viability of kidney epithelial Vero cells exposed to T-2 (0–120 nM) by the MTT assay. Similarly, 15 and 30 nM T-2 significantly decreased (*p* ≤ 0.05) the cell viability, while cells exposed to 7.5 nM T-2 did not show significant differences in cell viability with respect to the control. On the other hand, Martínez-Alonso et al. [27] studied the viability of human hepatocarcinoma HepG2 cells exposed to T-2 (7.5, 15 and 30 nM) and observed a cytotoxic effect with 30 nM T-2. However, these authors reported that 15 nM T-2 did not affect cell viability, and 7.5 nM T-2 significantly increased cell viability due to a possible hormetic effect in HepG2 cells.

### 2.4. Effects of a Simultaneous Treatment with T-2 and Tyrosol on Caco-2 Cell Viability

**Figure 5:**
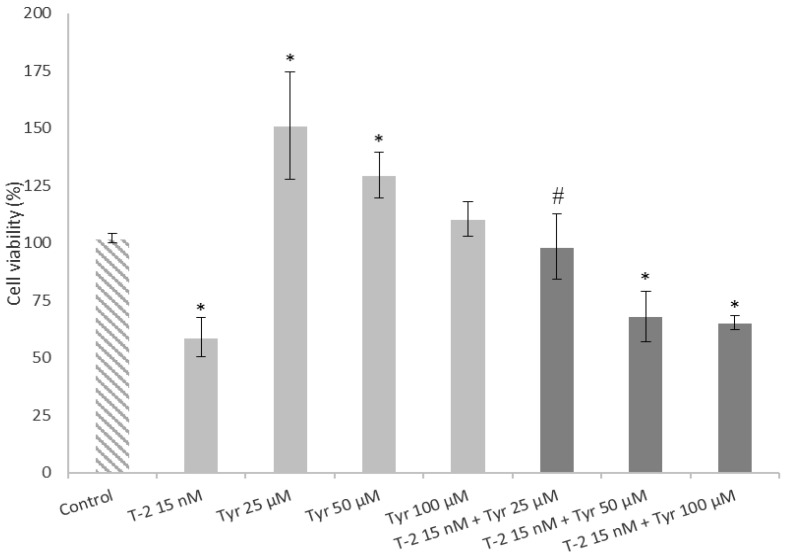
Effect of tyrosol (tyr) in Caco-2 cells exposed to T-2 in a simultaneous exposure. Cells were exposed to 1% DMSO (Control), T-2 15 nM, Tyr at 25, 50 and 100 µM or simultaneously exposed to T-2 and Tyr at each indicated concentration during 24 h. After 24 h of incubation, the viability of these cells was measured using the MTT assay. All values are expressed as mean ± SEM of 3 replicates. (*) *p* ≤ 0.05 indicates significant differences compared to control cells (medium with a final DMSO concentration ≤ 1%). (#) *p* ≤ 0.05 indicates significant differences compared to the results of Caco-2 cells exposed only to T-2.

With the aim to determine the cytoprotective effect of tyrosol in Caco-2 cells exposed to T-2, an MTT assay was performed. Caco-2 cells were simultaneously exposed to T-2 (15 nM) and tyrosol (25, 50 and 100 µM) (Figure 5). The viability of Caco-2 cells exposed to 25 and 50 µM tyrosol was significantly increased (*p* ≤ 0.05) with respect to the control by 49% and 27%, respectively. However, the highest concentration of tyrosol tested (100 µM) did not affect cell viability. These values are in agreement with the findings of Chiesi et al. [13] who reported a Caco-2 cell viability of 26% after exposure to 50 µM tyrosol.

On the other hand, incubation with 15 nM T-2 and 50 and 100 µM tyrosol decreased cell viability by 34% and 37% with respect to that of the control, respectively. On the contrary, 25 µM tyrosol prevented the toxic effects induced by exposure to 15 nM T-2, increasing cell viability compared to that of cells exposed only to T-2 (Figure 5). Similarly, Schaffer et al. [28] reported that low amounts of hydroxytyrosol prevented the oxidative stress-mediated loss of the MTT reduction potential in PC12 cells, a commonly used neuron-like cell culture model, whereas higher concentrations did not affect cell viability. On the contrary, no differences were observed in Caco-2 cell viability after simultaneous treatment with alternariol and tyrosol, and even the lowest concentration of tyrosol (25 µM) in combination with 100 µM of AOH produced a cell viability decrease with respect to alternariol alone [13].

### 2.5. Effects of a Pre-Treatment on the Viability of Caco-2 Cell Exposed to T-2 and Tyrosol

**Figure 6:**
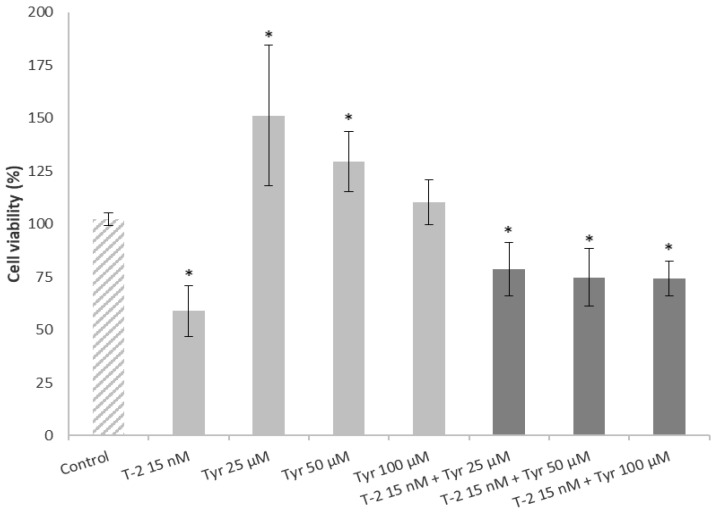
Effect of a pretreatment with tyrosol (tyr) in Caco-2 cells exposed to T-2. Cells were exposed to 1% DMSO (Control), T-2 15 nM, Tyr at 25, 50 and 100 µM concentrations or pretreated with Tyr at each concentration during 24 h and afterwards exposed to T-2 (15 nM). After 24 h of incubation, the viability of these cells was measured using the MTT assay. All values are expressed as mean ± SEM of 3 replicates. (*) *p* ≤ 0.05 indicates significant differences compared to control cells (medium with a final DMSO concentration ≤ 1%).

The effects of a cell pre-treatment with tyrosol (25, 50 and 100 M) during 24 h and followed by the addition of T-2 (15 nM) are presented in Figure 6. The findings indicated that a pre-treatment of Caco-2 cells with tyrosol was capable to decrease the cytotoxic effects caused by exposure to T-2. Nonetheless, Chiesi et al. [13] showed a cytoprotective effect when pretreating Caco-2 cells for 24 h with an EVOO extract before adding alternariol, a mycotoxin produced by *Alternaria alternata* spp.

On the contrary, Kössler et al. [29] showed that a long exposure time (22 h) to curcumin (phenolic compound) decreased the cell viability and induced apoptosis in a dose-dependent manner in human kidney cells (HEK293). Similarly, Martínez-Alonso et al. [27] indicated that exposure to a phenolic extract obtained from red beans reduced the viability of HepG2 cells in a time-dependent manner (24 h pre-treatment > 1 h pre-treatment), consistent with the results obtained in the present work.

## 3. Conclusions

The findings obtained in this study evidenced a bioaccessible fraction of T-2 of 51 ± 4% after in vitro gastrointestinal digestion of breakfast cereals when they were spiked with T-2 at 75 µg/kg. This bioaccessible fraction (diluted 1:16) did not affect Caco-2 cell viability after 24 h of exposure, due to the low concentration of T-2, quantified as 4.82 nM. On the one hand, the viability of Caco-2 cells exposed to T-2 (7.5, 15 and 30 nM) decreased in a dose-dependent manner, and T-2 showed significant cytotoxic effects at 15 and 30 nM. Conversely, tyrosol at 25 and 50 µM significantly increased cell viability, compared to the control, by 49% and 27%, respectively. Furthermore, the results obtained with the simultaneous treatment showed that a low concentration of tyrosol (25 µM) could attenuate the toxic effects induced by a low exposure to T-2 (15 nM) in Caco-2 cells, but a pre-treatment of Caco-2 cells with tyrosol did not prevent the cytotoxic effects caused by T-2. Nevertheless, more studies, such as the determination of oxidative stress (lipid peroxidation products or reactive oxygen species) and apoptosis levels in Caco-2 cells, are needed to corroborate this hypothesis. In addition, future research is needed to investigate if tyrosol could have an effect in vivo against the toxic effects produced by mycotoxins.

## 4. Materials and Methods

### 4.1. Reagents

The reagent-grade chemicals and cell culture components used, namely, Dulbecco’s Modified Eagle’s Medium (DMEM), trypsin/EDTA solutions, penicillin, streptomycin, 4-(2-hydroxyethyl)-1-piperazineethanesulfonic (HEPES), non-essential amino acids (NEAA), fungizone, phosphate-buffered saline (PBS), Fetal Bovine Serum (FBS), dimethyl sulfoxide (DMSO), methylthiazoltetrazolium salt (MTT), Sorensen´s glycine buffer, α-amilase, pancreatin, pepsin, bile salts, hydrochloric acid (HCl), potassium chloride (KCl), sodium hydroxide (NaOH), sodium chloride (NaCl), sodium phosphate dibasic (Na_2_HPO_4_), potassium phosphate monobasic (KH_2_PO_4_), sodium carbonate (Na_2_CO_3_), sodium bicarbonate (NaHCO_3_), ammonium carbonate ((NH_4_)_2_CO_3_), magnesium chloride hexahydrate ((MgCl_2_(H_2_O)_6_) and calcium chloride (CaCl_2_) were purchased from Sigma-Aldrich (Barcelona, Spain).

Methanol (MeOH) and water for the LC mobile phase (HPLC grade) were acquired from Merck (Darmstadt, Germany). Formic acid and ammonium formate were purchased from Fluka (Milan, Italy). Ethyl acetate was obtained from Merck (Madrid, Spain). Deionized water (resistivity < 18 MΩ cm) was obtained employing a Milli Q water purification system (Millipore, Bedford, MA, USA).

Standards of T-2 (≥98% purity) and tyrosol (≥98% purity) were purchased from Sigma Aldrich (Barcelona, Spain). Individual stock solutions of T-2 and tyrosol were prepared in DMSO at suitable working concentrations. The final concentrations of T-2 and tyrosol in the investigation were accomplished by adding the chemicals to the culture medium. The final DMSO concentration in the culture medium was ≤1% (*v*/*v*). Control cells were exposed to the same concentration of DMSO.

### 4.2. Sample Preparation Procedure

Breakfast cereals were acquired from a local supermarket located in Valencia, Spain. A sample (200 g) was homogenized with a grinder and stored in sterile plastic containers at room temperature. For the trials, blank samples (breakfast cereal previously analyzed to guarantee the absence of mycotoxins) and spiked samples (breakfast cereal spiked with T-2) were used. In this study, the spiked samples were obtained by adding T-2 at 75 µg/kg, the indicative levels being established by the European Commission for the sum of T-2 and HT-2 in breakfast cereals [8]. The spiked samples were stored overnight in darkness under refrigerated conditions. Later, each sample was subjected to an in vitro digestion method.

### 4.3. In Vitro Digestion Procedure

To determine the bioaccessible fraction of the T-2 toxin from breakfast cereals, the INFOGEST standardized in vitro gastrointestinal method was followed. Simulated salivary fluid (SSF), simulated gastric fluid (SGF) and simulated intestinal fluid (SIF) were obtained in accordance with Brodkorb et al. [30]. For the digestion, 2.5 g of previously diluted samples (standardized ratio of 1:1 (*w*/*w*) of sample and SSF) was taken, mixed with 2 mL of SSF and manually shaken for 1 min. Later, 0.75 mL of α-amylase solution (75 U/mL), 12.5 µL of 0.3 M CaCl_2_ and deionized water were added to a obtain a final volume of 5 mL. The mixture was placed in a shaking bath at 37 °C and 95 rpm during 2 min. Once the oral phase was completed, 4 mL of SGF, 0.5 mL of pepsin solution (2000 U/mL) and 5 µL of 0.3 M CaCl_2_ were added. Then, the mixture was mixed manually during 1 min. The pH of the mixture was adjusted to 3, and deionized water was added up to a final volume of 10 mL. The gastric mixture was incubated in a shaking bath for 2 h under similar conditions. To simulate the intestinal conditions, 4.25 mL of SIF, 2.5 mL of pancreatin solution (100 U/mL), 1.25 mL of bile salts and 20 µL of 0.3 M CaCl_2_ were added. The final mixture was stirred manually for 1 min and adjusted to pH 7, and deionized water was added to a final volume of 20 mL. At last, the mixture was incubated during 2 h at 37 °C and 95 rpm in a shaking bath. The digesta obtained were centrifuged (40 min, 4 °C, 4000 rpm), and the supernatant corresponding to the bioaccessible fraction was collected. All digestions were performed in triplicate. After each digestion step, aliquots of each phase were taken and stored in a freezer until mycotoxin determination. For the cell culture experiments, 200 μL/well of a suspension of Caco-2 cells at density of 2 × 10^4^ cells/well was placed in 96-well tissue culture plates.

### 4.4. Extraction Procedure

The extraction of T-2 from collected oral, gastric and intestinal samples was carried out through a liquid–liquid extraction using a method reported in the literature, with minor modifications [31]. In brief, 2 mL of each sample was placed into a 15 mL Falcon centrifuge tube, and then 2 mL of ethyl acetate was added, and the sample was vortexed for 1 min. The mixture was centrifuged for 3 min at 4000 rpm and 5 °C. Then, the supernatant phase was collected and evaporated to dryness under a gentle N_2_ stream at 45 °C with a TurboVap. The samples were reconstituted in 0.2 mL of a mixture of MeOH/H_2_O (70:30, *v*/*v*) and later filtered through a 0.22 µm filter, previous to their analysis by UHPLC-MS/MS.

### 4.5. UHPLC–MS/MS Analysis 

The samples were analyzed throughout a 1260 Infinity series UHPLC system (Agilent Technologies, Waldbrom, Germany) coupled to a triple quad 6500+ mass spectrometer equipped with an electrospray (ESI) Turbo V source (Sciex, Concord, ON, Canada). The software used for instrument control and data analysis was Analyst version 1.6.2. The column used was Waters Aquity BEH^®^ C18 Column (1.7 µm 100 Å, 50 × 2.1 mm, Waters, Milford, MA, USA) with, as mobile phase A, H_2_O with 0.1% formic acid and 5 nM ammonium formate and, as mobile phase B, MeOH with 0.1% formic acid and 5 nM ammonium formate. The gradient used was as follows: 0–2 min with 5% B, then linearly increased to 100% B for 13 min and held for 2 min. After that, B was decreased to 5% for 18 min and kept for another 2 min for column re-equilibration. The column temperature was set at 30 °C, whereas the flow rate was 350 µL/min, and the injection volume was 5 µL. Positive ionization mode was used for T-2 mass spectrometric detection, and selective reaction monitoring (SRM) was employed as scan type. The source settings were as follows: source temperature 300 °C, ion spray voltage 4.5 kV, curtain gas 30 psi, ion source gas 1 and 2 at 55 psi. SRM transitions were optimized by a syringe pump infusion of the T-2 solution. The UHPLC-MS/MS parameters are shown in Table 2.

### 4.6. Cell Culture and Treatment

Caco-2 cells were grown in DMEM medium supplemented with 10% FBS, 1% NEAA, 1% HEPES, 0.2% fungizone, 100 mg/mL streptomycin and 100 U/mL penicillin. This cell line was incubated in 5% CO_2_ at 37 °C, 95% humidity and pH 7.4. The cells were subcultured commonly twice a week, with a small number of sub-passages (<25 passages) so as to maintain genetic homogeneity. Caco-2 cells were subcultured after trypsinization with a 1:2 split ratio. The medium was replaced every 2–3 days. The final mycotoxin and polyphenol concentrations tested were accomplished by adding T-2 and tyrosol to the culture medium, with a final DMSO concentration ≤ 1% (*v*/*v*). The Central Service for Experimental Research (SCSIE) of the University of Valencia (Valencia, Spain) amiably supplied the Caco-2 cell line.

### 4.7. Determination of Cell Viability by the MTT Assay

A Caco-2 cell suspension (200 μL/well) at a density of 2 × 10^4^ cells/well was placed in 96-well tissue culture plates. Once the cells reached 80% confluence, the culture medium was replaced by fresh medium containing different concentrations of T-2 (7.5, 15 and 30 nM), tyrosol (25, 50 and 100 μM) or bioaccessible fraction (serial dilutions from 1:0 to 1:64). Then, the 96-well plates were incubated in darkness during 24 h at 5% CO_2_ and 37 °C. The three concentrations assayed were selected in agreement with preceding cytotoxic assays carried out elsewhere [32]. The concentrations of tyrosol were selected according to Chiesi et al. [13], who considered the consumption of olive oil by the Spanish population, which contains tyrosol as a major component. 

Two assays were carried out: a simultaneous treatment and a pre-treatment. On the one hand, for the pre-treatment studies, Caco-2 cells were exposed to tyrosol (25, 50 and 100 μM) for 24 h. Then, the medium containing tyrosol was removed, and the cells were exposed to T-2 (15 nM). On the other hand, to carry out studies with a simultaneous exposure, Caco-2 cells were simultaneously exposed to 7.5 nM T-2 and tyrosol (25, 50 and 100 μM) for 24 h. Three independent experiments for each assay were performed. The results are expressed as the mean ± standard error of the mean (SEM) of different independent experiments.

The MTT assay was employed to determinate Caco-2 cell viability. This assay is used to measure the cellular metabolic activity as an indicator of cell viability, proliferation and cytotoxicity. The MTT assay is based upon the ability of viable cells to metabolize MTT (a soluble yellow tetrazolium salt) to an insoluble purple formazan crystal. This assay was carried out following the procedure described by Ruiz et al. [32]. In brief, after the treatment studies, the medium containing the compounds was removed, and 200 μL of fresh medium containing 50 μL of MTT was added in each well. The 96-well plates were kept in the incubator at 37 °C and 5% CO_2_ during 3 h in darkness. After removing MTT solution, 200 μL of DMSO followed to 25 μL of Sorensen´s glycine buffer were added and mixed to dissolve the purple formazan crystals. The absorbance was read at 540 nm in an automatic ELISA plate reader (MultiSkanEX, Thermo Scientific, Walthman, MA, USA).

Cell viability was expressed in percent compared to that of control cells (≤1% DMSO). Three independent experiments were conducted, with eight replicates each. The results are expressed as the mean ± the standard error of the mean (SEM) of different independent experiments.

### 4.8. Statistical Analysis

SPSS version 214 (IBM Corp., Armonk, NY, USA) was used to perform the statistical analysis of the data. The data are expressed as mean ± SEM of different independent experiments. The statistical analysis of the results was carried out by the Student’s t-test for paired samples. Differences among groups were analyzed using one-way analysis of variance (ANOVA) followed by the Tukey HDS post-hoc test for multiple comparisons, and *p* ≤ 0.05 was considered statistically significant.

## Figures and Tables

**Table 1:** Percentage of T-2 released during gastrointestinal digestion of spiked breakfast cereals.

Digestion Phase	Sample	Released T-2 (%) (Mean ± SD; *n* = 3)
Oral Phase	Control	-
	Fortified ^1^	25 ± 8
Gastric Phase	Control	-
	Fortified ^1^	58 ± 7
Intestinal Phase	Control	-
	Fortified ^1^	51 ± 4

^1^ Fortification level (75 µg/kg).

**Table 2:** Parameters of UHPLC-MS/MS for the determination of the T-2 toxin.

Mycotoxin	Rt (min)	Precursor Ion	Product Ion	CE (eV)	DP (V)	EP (V)	CXP (V)
T-2	9.96	484.3	215.5	29	56	10	18
	9.96	484.3	185.1	31	56	10	11

Rt: retention time; CE: collision energy; DP: declustering potential; EP; entrance potential; CXP: collision cell exit potential.

## Data Availability

The data presented in this study are available on request from the corresponding author. The data are not publicly available to preserve the privacy of the volunteers that participated in the present study.
